# Male‐limited secondary sexual trait interacts with environment in determining female fitness

**DOI:** 10.1111/evo.13551

**Published:** 2018-07-19

**Authors:** Anna Maria Skwierzyńska, Jacek Radwan, Agata Plesnar‐Bielak

**Affiliations:** ^1^ Institute of Environmental Sciences Jagiellonian University Gronostajowa 7, 30‐387 Kraków Poland; ^2^ Institute of Environmental Biology, Faculty of Biology Adam Mickiewicz University Poznań Poland

**Keywords:** Alternative reproductive phenotypes, environment, fecundity, intralocus sexual conflict, male sexual characters, sexually antagonistic genetic variation, temperature

## Abstract

Selection for secondary sexual trait (SST) elaboration may increase intralocus sexual conflict over the optimal values of traits expressed from shared genomes. This conflict can reduce female fitness, and the resulting gender load can be exacerbated by environmental stress, with consequences for a population's ability to adapt to novel environments. However, how the evolution of SSTs interacts with environment in determining female fitness is not well understood. Here, we investigated this question using replicate lines of bulb mites selected for increased or decreased prevalence of a male SST—thickened legs used as weapons. The fitness of females from these lines was measured at a temperature to which the mites were adapted (24°C), as well as at two novel temperatures: 18°C and 28°C. We found the prevalence of the SST interacted with temperature in determining female fecundity. At 28°C, females from populations with high SST prevalence were less fecund than females from populations in which the SST was rare, but the reverse was true at 18°C. Thus, a novel environment does not universally depress female fitness more in populations with a high degree of sexually selected dimorphism. We discuss possible consequences of the interaction we detected for adaptation to novel environments.

Sexual selection arises through competition for mates and leads to the evolution of costly traits that facilitate this competition, such as weapons, aggressive behaviors, sexual ornaments, and displays to attract the opposite sex (Anderson 1994). These sexually selected traits (SSTs) are often sexually dimorphic, present only in the sex in which sexual selection is stronger (usually males). Despite this limitation, the genes underlying male SSTs can have pleiotropic effects on females. For example, in beetles and mites (Harano et al. 2010; Plesnar‐Bielak et al. 2014), artificial selection for an SST resulted in a correlated decline in female fitness. This demonstrates that SST evolution can contribute to gender load (Rice 1992; Arnqvist and Tuda 2010), that is the reduced fitness of one sex resulting from sexual conflict operating in a population. Gender load may originate from opposing fitness effects of an allele in the other sex–a situation referred to as intralocus sexual conflict (IASC) (Chippindale and Rice 2001; Bonduriansky and Chenoweth 2009). Intense research on IASC over the past two decades has demonstrated that it is pervasive among taxa and has implications for a wide range of fundamental evolutionary processes including the maintenance of genetic variation (Rice and Chippindale 2001; Connallon and Clark 2012; Rostant et al. 2015), speciation (Parker and Partridge 1998; Rice and Chippindale 2002), the evolution of gene expression (Ellegren and Parsch 2007) and of sex chromosomes (Rice 1984; Kraak and Pen 2002; van Doorn and Kirkpatrick 2007), sex allocation (Alonzo and Sinervo 2001), sexual selection (Pischedda and Chippindale 2006; Brommer et al. 2007), and aging (Vieira et al. 2000; Bonduriansky et al. 2008). Given their role in shaping gender load, the evolution of SSTs is likely to affect all of these processes.

However, recent evidence indicates that IASC can be modulated by the environment, so that the correlation between male and female fitness can sometimes change from being negative in a standard laboratory environment to becoming positive in a novel environment (Long et al. 2012; Berger et al. 2014a; but see Delcourt et al. 2009; Punzalan et al. 2014; Martinossi‐Allibert et al. 2018). This suggests that under natural conditions, characterized by environmental fluctuations, the role of sexually antagonistic genetic variation may be less pronounced than has been suggested by studies of populations in stable laboratory conditions (Long et al. 2012; Connallon and Hall 2016).

An increase in alignment between male and female fitness in novel environments would suggest that sexual selection should help, rather than hinder, adaptation. However, if gender load increases susceptibility to stress and affects a population's reproductive output, it would decrease the effective population size, thus hampering adaptation to environmental challenges and increasing inbreeding. These adverse effects may further increase a population's susceptibility to stress and force it into the extinction vortex (Gilpin and Soulé 1986) before it has a chance to adapt. For example, in seed beetles, genotypes that were associated with high male fitness but low female fitness at standard temperature had low fitness in both sexes in a novel, stressful environment (Berger et al. 2014a). Given the potential role of SSTs in causing gender load, their evolution is likely to affect female fitness when the environment changes, with consequences for rates of adaptation and population viability. However, pertinent empirical data are still lacking. Species that exhibit heritable, discontinuous variation in the expression of SSTs, such as many species that exhibit alternative reproductive phenotypes (which often accompany alternative reproductive tactics; Gross 1996; Oliveira et al. 2008) provide good systems with which to study IASC associated with SSTs; these models are particularly useful in that they can enable manipulation of SST frequency via artificial selection. Here, we investigated how the environment modulates female fitness in lines selected for the presence or absence of an SST in the male‐dimorphic bulb mite *Rhizoglyphus robini*.

In the bulb mite, two heritable male morphs occur (Radwan 1995). Fighter males are easily distinguished from females and the other male morph because their third pair of legs is thickened and sharply terminated, and is used to stab reproductive competitors (Radwan et al. 2000). Scrambler males, on the other hand, do not develop such a weapon and are not aggressive toward other males. Fighter males have been shown to achieve higher reproductive success than scrambler males in mixed‐morph populations (Radwan and Klimas 2001). However, scrambler phenotypes may be selected for indirectly, via selection acting on females: in a previous study, artificial selection on male morphs in replicated lines resulted in a correlated response in female fitness (Plesnar‐Bielak et al. 2014), despite the fact that females did not express the SST. Consistent with comparative evidence that increased sexually selected dimorphism is associated with elevated IASC (Cox and Calsbeek 2009), female fecundity and longevity were lower in replicate lines selected for the male fighter morph expressing the SST (the F‐lines) compared to lines selected for the male scrambler morph not expressing the SST (the S‐lines). At the same time, the morph identity of a female's partner did not influence fecundity, indicating that the observed conflict was indeed intralocus rather than phenotypic.

Here, we asked how the gender load associated with this SST varies across temperatures. Temperature is a key factor affecting ectotherm metabolism (Angilletta 2009), and morph‐specific and sex‐specific gene expression analyses have indicated that genes involved in metabolic processes are indeed associated with sexually antagonistic selection in fighters (Stuglik et al. 2014). Furthermore, Joag et al. (2016) found that selection for male morphs influenced the expression of over 500 genes (associated with a variety of organismal processes), and the expression profiles of these genes also significantly changed in females, indicating that widespread pleiotropic effects were associated with the male SST. If sexually selected phenotypes are more sensitive to the stress associated with a novel environment (Sheldon et al. 1998; Bussière et al. 2008; Berger et al. 2014a), then, owing to intersexual genetic correlations, female fitness should be more affected by the novel thermal environment in the F‐lines. However, it is possible that the effect of the SST on gender load will vary depending on the properties of the novel environment (Punzalan et al. 2014) or on the level of stress an environment imposes. To investigate the generality of any effect, we used two novel temperatures: higher and lower than the temperature to which the mites were adapted.

## Methods

### FEMALE FECUNDITY AND FERTILITY RATE

We used fighter‐ and scrambler‐selected lines (the F‐lines and the S‐lines, respectively) that had been selected for an increased proportion of one of the morphs for more than 120 generations (Plesnar‐Bielak et al. 2014). The lines have remained nearly monomorphic since generation 40. The selection was carried out in four replicates for each selection direction at 24°C. The populations from which the lines were derived had previously adapted to this temperature for over 10 years (more than 200 generations).

Each line was split into three temperature treatments, under which females developed, mated, and laid eggs: 18°C, 24°C, or 28°C. Our populations had not encountered 18°C or 28°C since they were moved to the lab, but both temperatures might be experienced by mites in their natural habitats in Poland. Indeed, 18°C is recorded at the ground surface quite often in spring and autumn, whereas 28°C occurs only occasionally in late spring and summer (when average surface temperatures vary between 20.07°C (SE = 10.45°C) in May and 25.2°C (SE = 10.24°C) in July; Ciaranek 2013). Earlier work revealed that 28°C is stressful for the mites, depressing female fecundity and interacting with low effective population size to cause extinction (Plesnar‐Bielak et al. 2012).

The experiment was initiated by collecting 60 previously mated females from each line and dividing them into three containers (20 females per container), where they laid eggs for 48 hours at 24°C. After this time, the females were discarded and the containers with eggs were randomly assigned to one of the three temperature treatments. When final‐instar nymphs emerged, 40–50 of them were collected from each container and placed into separate vials until adults emerged, at which point the adults were sexed; males were removed, and females were used in the fecundity assay.

For fecundity assays, each female was mated with a male from the stock colony. We used only scramblers, because they were more numerous in the stock colony and because a previous study had shown that the morph of a female's partner does not affect the number of eggs she lays (Plesnar‐Bielak et al. 2014). The males were kept at 24°C without access to females for 3 days before the assay to ensure they had sufficient sperm reserves for the duration of the experiment (so as not to constrain female fecundity). The pairs stayed together for 5 days at the appropriate treatment temperature, after which they were removed from the vials and the number of eggs laid by each female was recorded. A five‐day oviposition period can be treated as representative for a female's lifetime egg production, as the oviposition rate does not change significantly during the first three weeks of life, when females manage to lay the vast majority of their lifetime egg output (Konior et al. 2001; Tilszer et al. 2006). While lifespan, and hence the duration of egg laying, may vary between temperatures, our aim was not to compare lifetime egg output between temperatures, but to investigate how selection direction interacts with female fecundity rates. This interaction is unlikely to be affected by the effect of temperature on lifespan. Because the males were placed at the test temperatures for the duration of the assay, test temperature could have affected male fertility, which in turn might have contributed to differences in measurements of female fecundity. However, an auxiliary experiment we carried out showed that the temperature at which males were maintained did not influence the numbers of eggs laid by the females they mated with (Supplementary Material). Hence, all temperature effects we found in our study can be attributed to females.

The experiment was performed twice on females from generations 119 and 122 (block 1 and 2). Only the females that laid eggs were included in the analysis of fecundity. The numbers of females that laid eggs and those that failed to lay eggs were used as measures of female fertility rates.

### STATISTICS

The analyses were performed using R 3.3.3 (R Development Core Team 2017). The models were built using the lme4 package (Bates et al. 2015). Hypotheses were tested using the “ANOVA” function provided by the lmerTest package (Kuznetsova et al. 2016). We used type III sums of squares for the models including interaction, and type II sums of squares for the simplified model without interaction (Herr et al. 2016). The Kenward‐Roger approximation of degrees of freedom was used.

The data on female fecundity were analyzed using a linear mixed‐effects model. The number of eggs laid by each female was treated as the response variable. The model included temperature and selection direction as fixed factors, as well as their interaction. Block treated as a fixed factor and line identity (nested in selection direction) treated as a random factor were also included. Normality of residuals was inspected visually using Q–Q plots, and homoscedasticity of variance was examined by inspecting plots of residuals versus fitted values.

Female fertility or infertility was treated as a binomial response variable and analyzed using a generalized linear mixed‐effects model with a binomial distribution of errors. The model included temperature and selection direction as fixed factors, as well as their interaction. Block treated as a fixed factor and line identity (nested in selection direction) treated as a random factor was also included.

## Results

**Figure 1:**
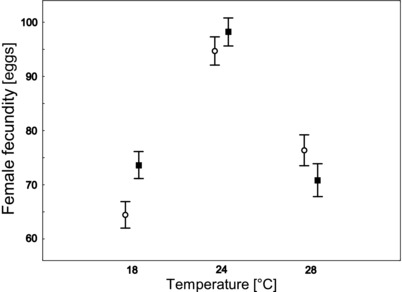
Mean fecundity of females from lines selected toward scramblers (the S‐lines, circles) and from lines selected toward fighters (the F‐lines, squares) measured at 18°C, 24°C, and 28°C. Bars denote standard errors.

**Figure 2:**
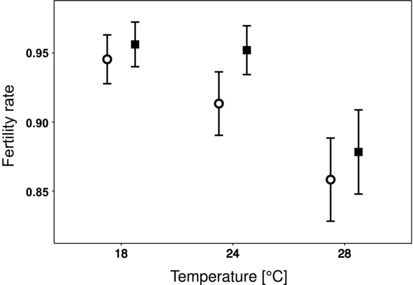
Fertility rates in lines selected toward scramblers (the S‐lines, circles) and lines selected toward fighters (the F‐lines, squares) measured at 18°C, 24°C, and 28°C. Bars denote standard errors.

We found that selection direction (the F‐lines vs the S‐lines) and temperature interacted significantly in their effect on female fecundity: females from the S‐lines were more fecund than females from the F‐lines at 28°C, but at 18°C the situation was reversed (Table 1; Fig. 1). Interestingly, contrary to expectation based on previous results (Plesnar‐Bielak et al. 2014), female fecundity in the S‐lines was not higher than that in the F‐lines at the control temperature, giving no indication of conflict between male fitness and female fecundity at that temperature (Fig. 1). At the same time, female fertility rates (proportions of fertile females) did not differ between selection directions at any temperature (Table 2). The removal of the nonsignificant interaction from the model showed that temperature, but not selection direction, influenced female fertility rates (temperature F_2, 862_ = 6.584; *P* = 0.001; direction of selection F_1,6_ = 1.646; *P* = 0.247); specifically, the proportion of infertile females was ca. two times higher at 28°C than at both 24°C and 18°C (Fig. 2).

**Table 1:** The effects of selection direction (toward fighters vs toward scramblers), temperature (18˚C, 24˚C, 28˚C), block, and line identity (nested in selection direction) on female fecundity, analyzed using a linear mixed‐effect model

	Fixed factors		
Effect	df	*F*	*P*
Selection direction	1;6	1.229	0.268
Temperature	2;794	64.641	<0.001
Selection direction × Temperature	2;794	3.711	0.025
Block	1;794	39.664	<0.001

	Random factors	
Effect	Variation explained	Standard deviation
Line identity	0.00	0.00

**Table 2:** The effects of selection direction (toward fighters vs toward scramblers), temperature (18˚C, 24˚C, 28˚C), block, and line identity (nested in selection direction) on fertility rates, analyzed using a generalized linear mixed‐effect model with a binomial distribution of errors

	Fixed factors		
Effect	df	*F*	*P*
Selection direction	1;6	1.359	0.288
Temperature	2;859	6.551	0.001
Selection direction × Temperature	2;859	0.307	0.736
Block	1;859	0.483	0.487

	Random factors	
Effect	Variation explained	Standard deviation
Line identity	0.00	0.00

## Discussion

We found that selection for or against an SST in males affected female fitness in an environment‐specific manner. This result is broadly consistent with the tenet of IASC theory that, because the sexes share genomes, selection on a male phenotype may affect female fitness. However, our results show that the nature of this relationship can be complex, with implications for several evolutionary processes, as we discuss below.

Previous work had demonstrated that selection for the SST in these lines resulted in a correlated decrease in female fitness (Plesnar‐Bielak et al. 2014), which is consistent with the widespread sexually antagonistic pleiotropy associated with the evolution of SSTs (Cox and Calsbeek 2009; Harano et al. 2010). Given the evidence for increased environmental sensitivity in sexually selected phenotypes (Sheldon et al. 1998; Bussière et al. 2008) and in sexually antagonistic genotypes (Berger et al. 2014a), we predicted that novel environments would exacerbate the difference in fitness between the F‐line and S‐line females in favor of the latter. However, we observed a crossover interaction, with the F‐line females faring worse at increased temperature (28°C, as predicted), but better at the decreased temperature (18°C, counter to the prediction). Their lower fitness at the increased temperature was in line with the results of Berger et al. (2014a), who showed that isofemale lines of seed beetles that harbored genotypes associated with high male reproductive competitiveness but low female fitness performed poorly in a stressful high‐temperature environment. These authors suggested that this could be due to an association between high fitness in males and high metabolic rates, which could become detrimental at high temperatures given that temperature increases metabolic rates in a deterministic manner. Our results are also consistent with those of Plesnar‐Bielak et al. (2013), who observed a decline in the proportion of fighter males in bulb mite populations evolving at 28°C. While the effect of temperature on the relative fitness of male morphs remains to be investigated, our results demonstrate that genes associated with fighter morphs can be selected against at 28°C when expressed in females.

However, in contrast to what we observed at 28°C, female fecundity in the F‐lines was higher than that in the S‐lines at 18°C. This highlights that the costs and benefits to females of expressing genes that are sexually selected for in males may vary across environments. These costs and benefits might depend on the level of stress imposed by a given environment. While a similar decline in the rate of egg laying was observed in our study for both novel temperatures (Fig. 1), fertility rates were only affected at the increased temperature (Fig. 2). Furthermore, the lifespan of bulb mites has been shown to increase at decreased temperature (Plesnar‐Bielak et al. 2018), so that at 18°C the oviposition period is probably extended with respect to that at both 24°C and 28°C. Hence, while both 18°C and 28°C can be considered novel environments for our populations, the increased temperature might in fact be more stressful than the decreased temperature. Our results could suggest that the negative pleiotropic effects of the male SST on female fitness were exacerbated in highly stressful environments such as 28°C, but not in environments in which the stress was milder. This could be explained by the higher sensitivity of sexually selected phenotypes to stress (Berger et al. 2014b), coupled with the pleiotropic effect of SSTs on female fitness (Plesnar‐Bielak et al. 2014; Joag et al. 2016). Another interpretation of our results is that whether gender load is increased or decreased in a novel environment depends on the precise properties of a given environment rather than on the magnitude of stress it imposes on a population (see also Delcourt et al. 2009; Punzalan et al. 2014). In our study, the level of SST‐mediated IASC may be associated with metabolic costs in females due to the expression of genes associated with the fighter phenotype; such costs are probably increased at higher temperature, but decreased at lower temperature (in line with the results of Berger et al. 2014a).

The finding that, for females, the costs and benefits of expressing male‐SST‐associated genes are environment‐specific can have implications for adaptation to novel environments. Theory predicts that sexually antagonistic pleiotropy tends to maintain genetic variation in populations (Fry 2010; Connallon and Clark 2012). On that premise, it has been speculated that sexual selection, by enhancing the potential for IASC, could increase the adaptive potential of populations by helping to maintain variation at ecologically relevant genes (Radwan et al. 2016). The presence of an SST in a population may cause a gender load in an environment to which the population is adapted—maintaining alleles that benefit males and harm females—but such alleles may sometimes prove beneficial to females when the environment changes. Our finding that the fitness of F‐line females was increased at the novel temperature of 18°C is in line with this prediction.

In contrast to previous findings (Plesnar‐Bielak et al. 2014), we found no evidence for IASC in the fighter‐selected lines at 24°C. One explanation is that the IASC reported by Plesnar‐Bielak et al. (2014) in two independent experiments may have been resolved in the generations intervening between that study (carried out after 55 generations of artificial selection) and the present one (generation 120). This seems plausible given that resolution of sexual conflict within a similar timeframe was reported for *D. melanogaster* (Collet et al. 2016). Clearly, though, the hypothesis that sexual conflict has been resolved at the standard temperature of 24°C should be tested further. One prediction would be that the previously reported correlation in gene expression patterns between fighter males and females (Joag et al. 2016), which was thought to underlie the increase in IASC, has weakened.

Under natural conditions, when two morphs co‐occur, sexual antagonism may be hard to resolve because alleles that are beneficial for females but detrimental for males when expressed in one morph (e.g., fighters), may be neutral or beneficial in another morph (e.g., scramblers), weakening or opposing selection for sex‐specific gene expression. Spatio‐temporal variation in temperature, which can reverse the effects of morph‐specific genes on female fitness, can further weaken selection for conflict resolution (Connallon and Hall 2016). Overall, our results suggest that complex interactions between sexual conflict, environment, and within‐sex phenotypic variation can have important consequences that deserve to be further explored.

Associate Editor: J. McKinnon

Handling Editor: Mohamed A.F. Noor

## Supporting information


**Fig. S1**. Mean fecundity of females developed and maintained at 24°C or 28°C following mating with males maintained at 24°C (squares) or 28°C (circles).
