# Supplementary material for: Male‐limited secondary sexual trait interacts with environment in determining female fitness
Source: Evolution. 2018 Jul 19;72(8):1716–22. doi: 10.1111/evo.13551 (PMC6175437; doi:10.1111/evo.13551)
Supplement: Supplementary file 1 — Fig. S1. Mean fecundity of females developed and maintained at 24°C or 28°C following mating with males maintained at 24°C (squares) or 28°C (circles). [file EVO-72-1716-s001.docx]

**Methods**

We ran an additional experiment to rule out the possibility that the temperature effects on female fecundity found in the main experiment were to some extent mediated by the effect of temperature on their male partners during the mating period. We compared fecundity and fertility of females mated to males that were reared at either 24°C or 28°C during the mating period. These two male treatments were crossed with analogous female treatments. Except for the full factorial design of male and female temperature treatments, all experimental procedures replicated the main experiment as closely as possible.

We took 250 males from the stock colony (kept at 24°C) and put them into individual vials for three days to let them restore their sperm supply. After this time, the males were placed at either 24°C or 28°C for the next six days (they were assigned to the thermal treatments randomly). Then, each male was assigned to a virgin female that had developed at either 24°C or 28°C. We established 45 mating pairs for each combination of female and male rearing temperatures (180 pairs in total).

The mating assay lasted for five days. Each day the pairs were allowed to mate for 3 hours at 26°C; following this, they were separated and returned to their original rearing temperature for 21 hours, until the next day’s mating session. The mating temperature of 26°C was chosen as the intermediate between 24°C and 28°C, so as to both minimize and standardize stress associated with temperature changes for the mated individuals. After the fifth mating session, males were discarded and females were moved to new containers for an additional five days of oviposition. We counted eggs laid by females during both the mating periods and the oviposition period. Pairs in which any of the individuals died before the end of the mating period (25 observations in total) were removed from the analysis. The fecundity analysis included only pairs that produced eggs (9 pairs were removed from this analysis, but they were included in the fertility rate measurement).

Fecundity (the number of eggs laid by females) was analyzed using an analysis of variance with female rearing temperature, male rearing temperature, and their interaction as factors. Normality of residuals was inspected visually using Q-Q plots, and homoscedasticity of variance was assured by inspecting plots of residuals versus fitted values. We used type III sums of squares for the initial model that included the interaction, and type II sums of squares for the simplified model without the interaction (Herr *et al.* 2016). The Kenward-Roger approximation of degrees of freedom was used.

Fertility or infertility was treated as a binomial response variable (fertile pairs were coded as 1 and infertile as 0) and analyzed using a generalized linear model with a binomial distribution of errors. The model included female rearing temperature, male rearing temperature, and their interaction as factors.

**Results**

Female rearing temperature significantly influenced the number of eggs laid (F_1,143_ = 89.557; p < 0.001; Fig. S1), with females originating from the standard temperature being more fecund than those from the increased temperature. Male temperature, however, had no effect on the fecundity of the female partners (F_1,143_ = 1.722; p = 0.192). The interaction of male and female temperatures was insignificant (F_1,143_ = 0.076; p = 0.783), and removing this insignificant interaction did not qualitatively change the result (female temperature F_1,144_ = 90.136; p < 0.001; male temperature F_1,144_ = 1.733; p = 0.190).

Neither female (F_1,151_ = 1.292; p = 0.256) nor male rearing temperature (F_1,151_ = 0.323; p = 0.571) influenced fertility rates. The interaction of male and female rearing temperatures was also insignificant (F_1,151_ = 0.242; p = 0.626), and removing this insignificant interaction did not qualitatively change the result (female temperature F_1,152_ = 1.191; p =0.277; male temperature F_1,152_ = 0.191; p = 0.663).

**Conclusion**

We did not find any evidence that the temperature at which males had been maintained affected the number of eggs laid by their female partners. This justifies our attribution of the temperature effects found in our main experiment to females from the selection lines rather than to thermal effects on male fertility.


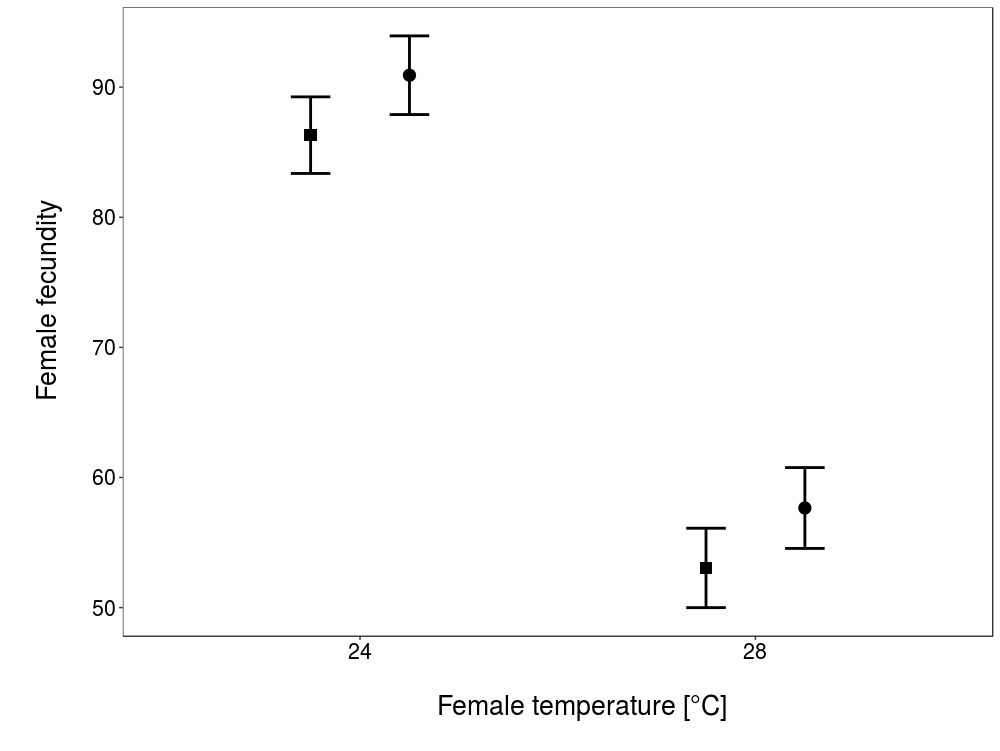


Figure S1. Mean fecundity of females developed and maintained at 24°C or 28°C following mating with males maintained at 24°C (squares) or 28°C (circles). Bars denote standard errors.
